# Self-Assembled Monolayers of Alkanethiols on Nickel Insert: Characterization of Friction and Analysis on Demolding Quality in Microinjection Molding

**DOI:** 10.3390/mi12060636

**Published:** 2021-05-29

**Authors:** Jiachen Chen, Jin Yang, Mingyong Zhou, Can Weng

**Affiliations:** College of Mechanical and Electrical Engineering, Central South University, Changsha 410083, China; csuchenjiachen@csu.edu.cn (J.C.); yj20141116@csu.edu.cn (J.Y.)

**Keywords:** microstructure, injection molding, self-assembled monolayers, alkyl mercaptan

## Abstract

When the part geometry scaling down from macro to microscale level, the size-induced surface effect becomes significant in the injection molding process. The adhesion between polymer and nickel (Ni) mold insert during the process can lead to defects in necking, warping and deformation of microstructure. In this study, the self-assembled monolayers (SAMs) with low surface energy were deposited on the Ni surface to reduce the adhesion and further improve the demolding quality of the microstructure. Results show that the alkyl mercaptan SAMs with chemical bonds and close alignment can be successfully deposited on the surface of Ni by the solution deposition method. The contact angle, surface free energy, and friction coefficient before and after anti-adhesion treatment on the surface of mold insert were measured. In addition, the anti-adhesion properties of different alkyl mercaptan materials and the correspondingly replication quality of microstructure parts after injection molding were analyzed. It is found that the Ni mold insert treated by the perfluorodecanethiol has the best wear resistance and still shows good reproducibility at the 100th demolding cycle.

## 1. Introduction

Injection molding technology is a common method to fabricate plastic products in biomedicine [1,2,3], chemical detection [4,5], optical security [6,7], and photoelectric technology [8,9] with complex shape and size [10,11,12]. However, the process of demolding will directly affect the replication quality of the micro/nano structures [13,14,15].

When the size of the structure is down to micrometer or nanometer, microscale factors including the mold surface roughness may obviously affect the adhesion characteristics of the interface between polymer and mold insert [16]. Stormonth-Darling et al. [17] studied the deformation of polycarbonate nanopillars during the demolding process. It was demonstrated that the elongation of the nanostructure was caused by the strong adhesion and friction between the top of the nanopillar and the nano-cavity. The interaction between polymer and insert interface would affect the demolding stage [18]. The higher the surface roughness of the mold insert, the greater the adhesion between the polymer and the mold insert, which leads to higher demolding resistance. Sasaki et al. [19] studied the relationship between the demolding resistance of different polymer materials and the surface roughness of the mold insert. When the surface roughness of the insert was greater than 0.1 μm, the demolding resistance was mainly determined by the friction of the polymer-insert interface. When the surface roughness was less than 0.1 μm, the main factor affecting the demolding resistance was the van der Waals force, that is, the non-bonding interaction between the metal insert and polymer material.

In order to eliminate the demolding defects in injection molding process, Douglas et al. [20] treated the polycrystalline silicon microdevices with fluoro-octyltrichlorosilane (FOTS) self-assembled monolayers (SAMs). The experimental results showed that the adhesion increased, and the adhesion energy decreased logarithmically with the increase of impact cycles. These results provided a basis for studying the physical and chemical changes of SAMs surface with low surface energy. Kwon et al. [21] studied the effect of FOTS SAMs on the imprinting quality by molecular dynamics method. The self-assembled alkanethiols is also an alternative in anti-adhesion application, since the alkanethiol monolayers could be effectively adsorbed on metal surface. Experimental methods were utilized to deposit thin SAMs on the metal surface to reduce the friction and adhesion. Bowen et al. [22] introduced the long-chain methyl end chain alkyl mercaptan as a SAMs layer due to the exhibit wetting behavior and low surface energy. It could be assumed that surface lubricity plays a decisive role in surface adhesion for that. In order to improve the poor adhesion of polymer coatings, Chen et al. [23] used polytetrafluoroethylene and zirconia low heat transfer coefficient mold coating, and the surface roughness of microporous parts was used to evaluate the appearance quality of microporous parts. The numerical and experimental results showed that the two kinds of mold coatings could achieve the delayed heat transfer between the melt and the mold, improve the fluidity of the melt and effectively reduce the shear force of the mold on the melt. Yooh et al. [24] studied the effect of depositing FOTS SAMs on the surface of silicon on the injection molding of microstructure array parts and measured the contact angle to reflect the surface energy. The results showed that the SAMs could reduce the adhesion between the polymer and the silicon.

It is demonstrated from the previous studies that the deposition of appropriate coatings on metal or nonmetal materials can effectively reduce the adhesion between interfaces and improve the performance [16,25,26]. However, the tribological properties and durability of the SAMs on a certain metal surface have not been comprehensively studied. The studies of mold insert with SAMs coatings during the demolding process are less reported. In this paper, different alkyl mercaptan solutions were prepared and deposited on the Ni mold insert for injection molding. The quality, morphology and friction properties of the microparts were compared by injection of the Ni insert treated with different anti-adhesion SAMs. The morphology and physical properties of the SAMs of alkyl mercaptan were measured by the atomic force microscope (AFM), contact angle meter and friction wear tester. The surface microstructure array was characterized by the laser confocal microscope.

## 2. Materials and Methods

### 2.1. Fabrication of Mold Insert

**Figure 1:**
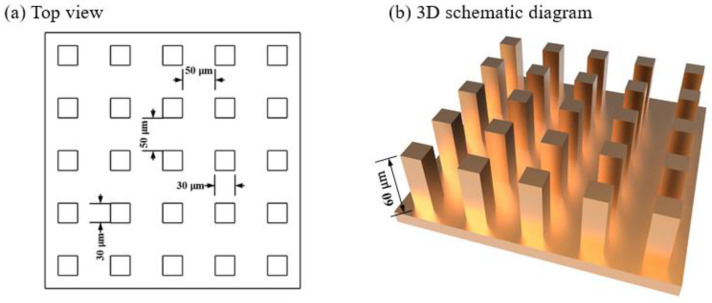
The main dimensional parameters of surface microstructure: (**a**) top view; (**b**) 3D schematic diagram.

The microstructure with quadrangular column arrays was designed in this study [27]. The aspect ratio was 2:1 and parameters were shown in Figure 1.

**Figure 2:**
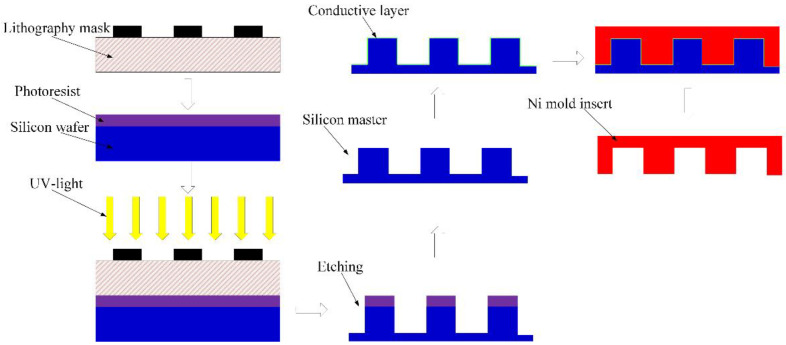
Manufacturing process of the Ni mold insert.

Due to the excellent mechanical properties, Ni was selected as the insert material for the injection molding of the parts with microstructure arrays [28]. Figure 2 is the flow chart of the fabrication of mold inserts for designed microquadrangular arrays. Silicon master was prepared by UV lithography (URE-2000135, Chengdu, China) and followed by the reactive ion etching (ULVAC NLD 570, Kanagawa, Japan). 

**Figure 3:**
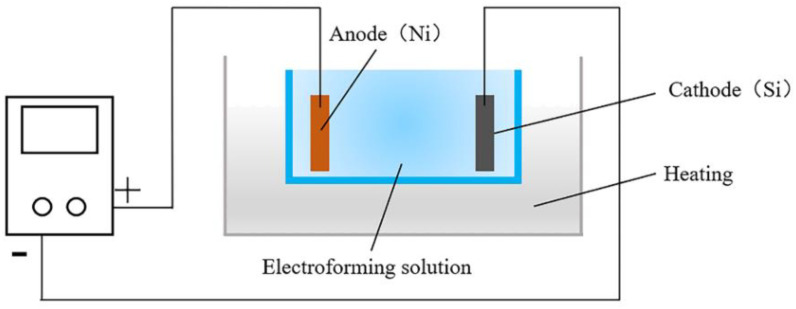
The V-30L micro-electroforming device.

A self-designed electroforming device (V-30L, Changsha, China), as shown in Figure 3, was used to fabricate the Ni mold insert [29]. Sputtering coater (Leica EMSCD500, Wetzlar, Germany) was used to conduct the silicon master by depositing a gold layer on the surface, so that the metal ions will be effectively transferred and deposited on the silicon master in the later electroforming process. The Ni sulfamate was selected as the electroforming solution with the back plate method during the process. In order to ensure a sufficient filling of the microstructure, a small current density of 1 A/dm^2^ was used for the first 30 min. The current density was increased to 4 A/dm^2^ for 20 h to improve the electroforming efficiency. The Ni coating was then electroformed as the mold insert for the following injection molding process. 

### 2.2. SAMs Preparation

The reagents used in the preparation of anti-adhesion SAMs were decanethiol (DT), decanedithiol (DDT) and perfluorodecanethiol (PFDT). The basic attribute parameters are shown in the Table 1.

**Figure 4:**
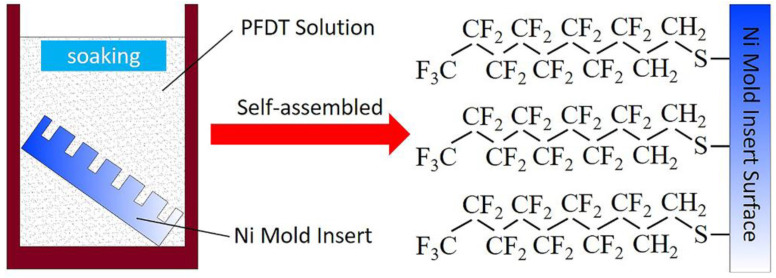
Self-assembled PFDT on the surface of Ni mold insert.

In the preparation of SAMs, the Ni mold insert was immersed in the solution containing the self-assembled molecule. The active groups of organic molecules would spontaneously adsorb on the surface to form the dense and ordered SAMs. The self-assembly of Ni mold insert in PFDT solution was shown in Figure 4. The solution deposition method [30,31] can make the sulfur atoms in the head of alkyl mercaptan molecules form chemical bonds with the Ni atoms, thus forming two-dimensional monolayers which were connected by chemical bonds and closely aligned on the Ni surface.

Prior to that, the Ni mold insert was pre-treated to remove the grease, organic and inorganic impurities in an ultrasonic cleaner 15 min. The insert was then treated with the plasma cleaning device (SunJune VP-R3, Guangzhou, China) for 200 s to remove the oxide and generate the hydrophilic groups that was conducive to the deposition of alkyl mercaptan molecules on the Ni surface. Benzene was selected as the solvent in this experiment, as this non-polar solvent will not react with metal matrix. Therefore, 20 mL benzene was mixed with three kinds of alkyl mercaptan reagents individually, with the concentration of 0.1 mol/L. After the alkyl mercaptan solution was magnetic stirred at room temperature for 3 h, the pre-treated insert was immersed in alkyl mercaptan solution for 24 h. Finally, the insert in the alkyl mercaptan solution was taken out, washed with deionized water, and then blown dried at 100 °C for 30 min. The drying process was conducive to enhancing the adhesion between the self-assembled molecules and the mold insert. 

### 2.3. Injection Molding

The precision injection molding machine (Sodick TR05EH2, Kanagawa, Japan) was used to prepare the polymer parts with surface quadrangular column array. During the experiment, the mold temperature controller (Yanbang YBM-1H, Dongguan, China) and the cooling system (Xinyi Electric SIC-3A, Zhongshan, China) was used to control the temperature of the mold and the injection machine. Polypropylene (PP, HD120MO, Borealis AG, Vienna, Austria), with excellent filling performance, high mechanical strength and melt flow rate, was selected as the experimental material. The prepared Ni mold insert was installed in the fixed mold cavity, and the effect of anti-adhesion treatment on the demolding quality of the microstructure was explored. The standard process parameters were used to study the demolding quality of polymer parts by comparing the quality without anti-adhesion treatment and after alkyl mercaptan treatment, as shown in Table 2 on the surface of Ni mold insert. A total of four groups of experiments were conducted, and each group of experiment was repeated for 20 cycles. 

### 2.4. Chemical and Structural Characterization

The laser confocal microscope (Carl Zeiss LSM 700, Oberkochen, Germany) was used to analyze the morphology of the microstructure on both mold insert and the replicated products.

The AFM (Veeco NanoManVS/Multimode, Santa Barbara, CA, USA) in tapping mode was used to measure the morphology of the SAMs, with the scanning range of 1 μm × 1 μm. 

Contact angle meter (Baioulin Theta, Gothenburg, Sweden) was used to measure the static contact angles. The droplet was set to 4 μL in each test. The contact angle of water droplets on the Ni surface was affected by the small temperature change, which was the cause of the error. In addition, 120 measurements of each sample were performed in the measurement of contact angle.

The friction and wear properties of SAMs on the insert surface were measured by the precision friction and wear tester (Anton Paar TRB3, Graz, Austria) at room temperature. The equipment can be applied with a loading range of 1–20 N, and friction mode is ball/pin disc. The friction pair was a standard Si_3_N_4_ ball, with a diameter of 6 mm. The normal load, the radius of wear track and the linear sliding speed were 1 N, 2.5 mm and 5 mm/s, respectively, and the rotation angular speed was 60 rpm. The surface of each sample was tested for at least three cycles, and each test was rubbed for 100 cycles.

## 3. Results and Discussion

### 3.1. Quality Analysis of Mold Insert

**Figure 5:**
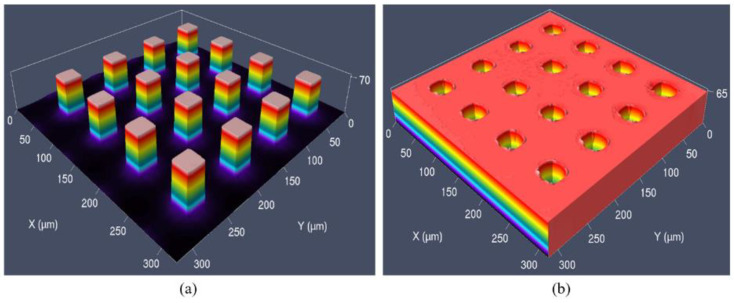
The microstructure arrays on both (**a**) Si master; (**b**) Ni mold insert by the confocal microscope.

The microstructure arrays on Si master and the Ni mold insert manufactured in this study were shown in Figure 5. It is seen that the electroformed microstructure was uniformly replicated with high homogeneity. The average size of microcolumn on Si master was 26.42 μm in width and 54.11 μm in height, while the microcavity on Ni mold insert was 23.19 μm in width and 60.66 μm in depth. This acceptable error might be the shrinkage of the microcavity caused by the release of residual stress in the Ni mold insert after electroforming.

**Figure 6:**
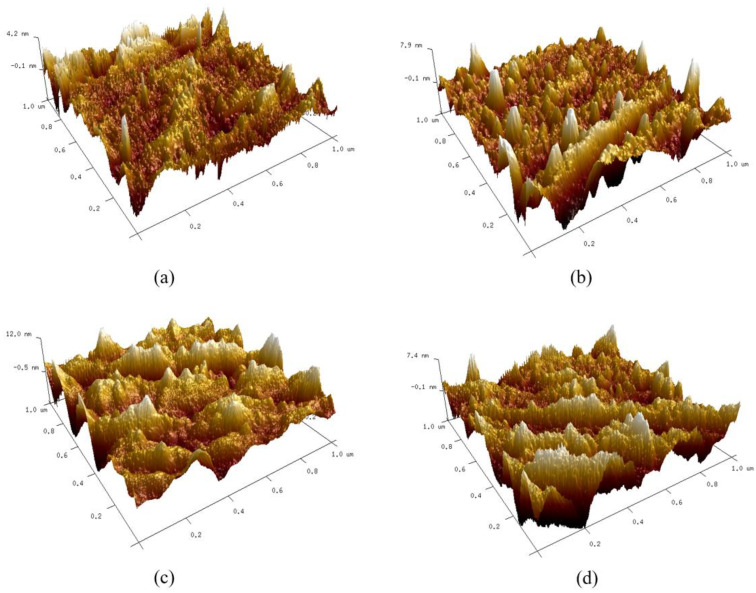
Three-dimensional morphology on the surface of mold insert: (**a**) no coating; (**b**) DT; (**c**) DDT; (**d**) PFDT.

The three-dimensional morphology and surface roughness with no coating and the SAMs with different alkyl mercaptans were shown in Figure 6 and Table 3 respectively. It could be seen that the Ni surface with no coating had less bumps and no self-assembled single molecule, and its surface roughness was less than that of the other three SAMs. It is seen from Figure 6b–d that the surface of Ni was rough and there were many columnar protrusions on the surface, indicating that alkyl mercaptan molecules had been successfully deposited on the Ni surface. The surface roughness of the SAMs of DT, DDT and PFDT were 1.35, 2.78 and 1.69 nm, respectively. The surface roughness of DDT and PFDT SAMs was higher than that of DT SAMs.

### 3.2. Contact Angles Measurement

**Figure 7:**
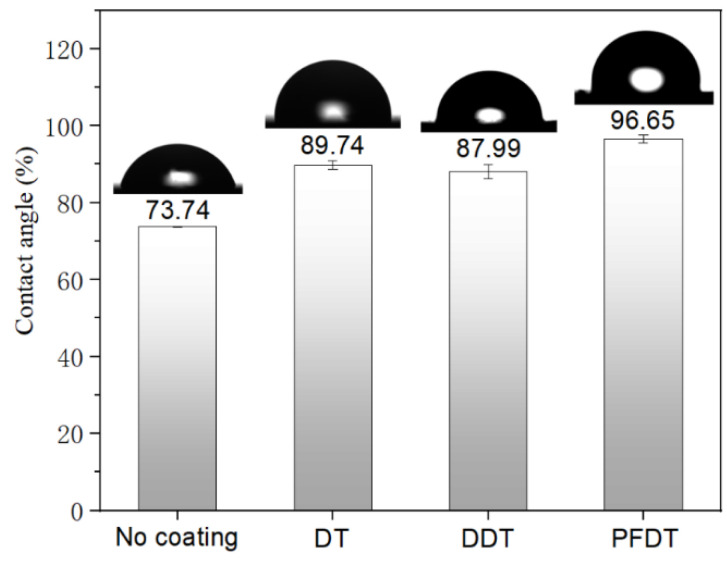
Contact angles of different alkyl mercaptan SAMs deposited on the insert surface.

The static contact angles of three different positions were measured with water drops on each surface, and the average contact angles of three sampling points were taken. The contact angles of Ni surface treated with different alkyl mercaptans were shown in Figure 7. 

The static water contact angle of the surface of Ni insert was 73.74°. However, the static water contact angle increased significantly after deposition of alkyl mercaptan SAMs, indicating that alkyl mercaptan self-assembly molecules were successfully deposited on the surface of Ni mold insert, which made the contact angle of Ni mold insert surface increased.

### 3.3. Friction and Wear Test

**Figure 8:**
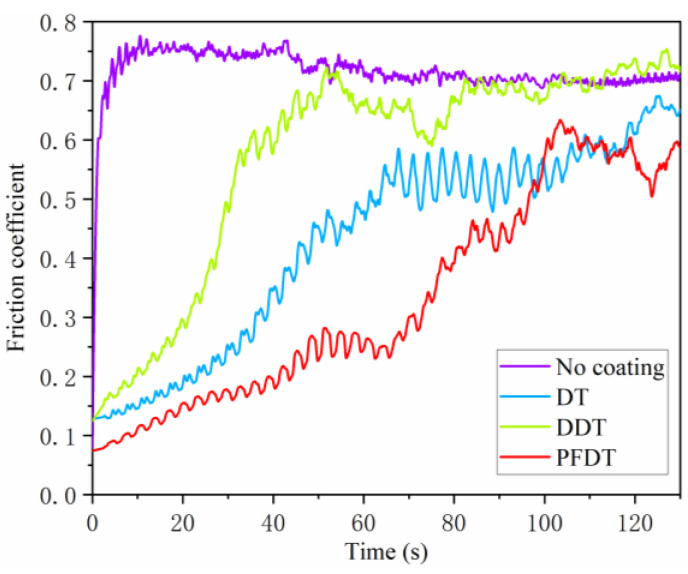
Friction coefficient of SAMs on Ni surface with the friction time.

The friction coefficient on Ni surface was shown in Figure 8. After 10 s, the friction coefficient of Ni surface with no coating increased sharply and reached the peak value of 0.77. The friction coefficient was relatively low due to the dry friction between the Si_3_N_4_ ball and the oxide on the Ni surface that acted as the lubricant [32]. The friction coefficient decreased slightly and fluctuates around 0.70 afterwards. On the other hand, the friction coefficient of Ni surface with self-assembled alkyl mercaptan was much lower, which would be due to the lower surface free energy of Ni surface with the SAMs [33]. It is demonstrated that the alkyl mercaptan SAMs had a good lubricity and could effectively reduce the friction coefficient. 

In the first 20 s of friction test, the friction coefficient of Ni surface with DT SAMs was less than 0.2. The friction coefficient then increased slowly to 0.53 and fluctuated with the increase of time. It means that DT SAMs have been worn through by Si_3_N_4_ ball. The SAMs lost their lubricating effect and showed dry friction with Si_3_N_4_ ball. Therefore, the wear resistance of DT SAMs was poor. In the whole friction process, the friction coefficient of DDT SAMs was much higher than that of DT SAMs. After 40 s, the friction, Si_3_N_4_ ball almost wore through DDT SAMs. At the later stage of friction, dry friction occurred between Si_3_N_4_ ball and Ni surface, and the friction coefficient fluctuates around 0.7, which was basically the same as that of Ni surface without SAMs. The friction coefficient of PFDT SAMs was lower than that of the other two SAMs at 100 s, and the SAMs began to wear rapidly after 60 s. The results showed that the PFDT SAMs had a good wear resistance and good lubricity in early time, which was due to the low surface energy of trifluoromethyl in PFDT. 

### 3.4. Analysis of Demolding Quality

**Figure 9:**
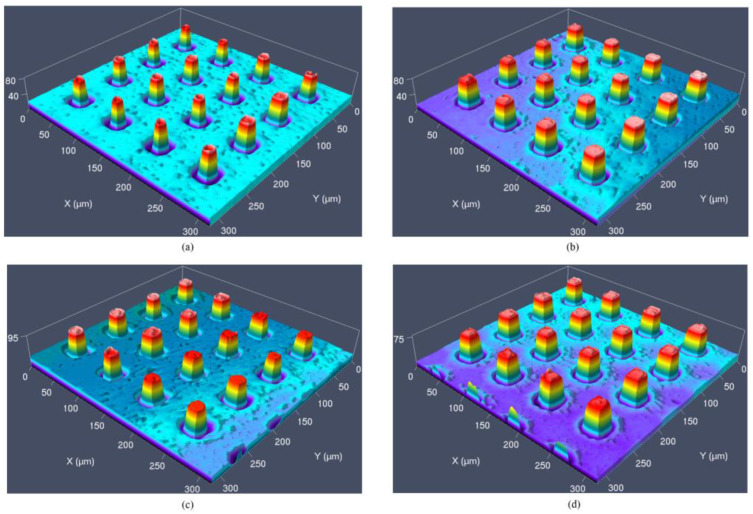
Three-dimensional topography after first demolding of (**a**) no coating; (**b**) DT; (**c**) DDT; (**d**) PFDT.

The injection-molded product after the first demolding was selected to observe the microstructure by the laser confocal microscope. The three-dimensional morphology of the microcolumn arrays was shown in Figure 9, which could show the demolding effect of the modified surface on Ni mold insert. 

**Figure 10:**
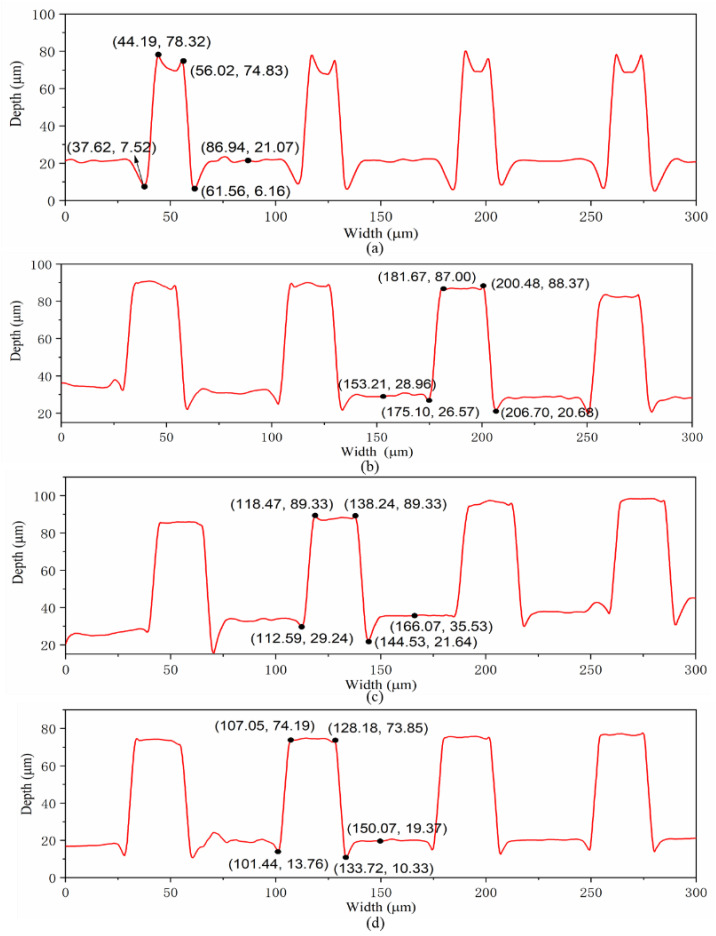
Profiles of microstructure after the first demolding by the laser confocal microscope: (**a**) no coating; (**b**) DT; (**c**) DDT; (**d**) PFDT.

The cross-section of a microcolumn was intercepted, and the profile curve was shown in Figure 10. It could be found that when the SAMs were not deposited on the surface of the Ni mold insert, the width of the microcolumn was relatively small, and most of the microcolumns were not fully replicated. However, the morphology and plumpness of the microcolumn were obviously improved after the alkyl mercaptan was deposited on the surface. Therefore, the microstructure of the microcavity in the mold insert could be well reproduced, and the microcolumn could be successfully demolded from the Ni mold insert with better quality.

In order to quantitatively analyze the demolding quality of microcolumn, the deviation percentage in height and width of the profiles were defined to describe the deviation between the profile of the injection-molded microstructure and the microcavity in Ni insert. The calculation formulas were as follows:

(1) $$a_{h}=\frac{H_{c}-H_{m}}{H_{m}}\times 100\%$$

where $a_{h}$, $H_{c}$ and $H_{m}$ were the height deviation percentage, the average of left and right height of micron column and these of microcavity, respectively.

(2) $$\beta_{w}=\frac{W_{c}-W_{m}}{W_{m}}\times 100\%$$

where $\beta_{w}$, $W_{c}$ and $W_{m}$ were the width deviation percentage, the average of upper and lower width of micron column and these of microcavity, respectively.

**Figure 11:**
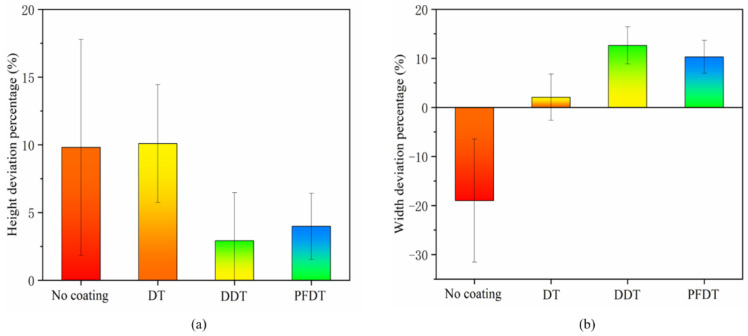
Deviation percentages of the microstructure: (**a**) height; (**b**) width.

In total of fifty microcolumns were randomly selected to calculate the deviation percentage. The calculation results were shown in Figure 11a,b.

It is shown that the height deviation of microcolumn profile was positive, which indicated that microcolumn had an elongation deformation after demolding from Ni mold insert under all conditions. When the DDT and PFDT SAMs were deposited on the surface of the mold insert, the height deviation of the microcolumn profile was relatively small. When there was no self-assembled coating on the insert surface, the width deviation was negative, which indicated that the microstructure has a shrinkage deformation. When the alkyl mercaptan layer was deposited on the insert surface, the width deviation was positive, which indicated that the microstructure had expansion deformation.

### 3.5. Duration Analysis of Mold Insert with SAMs

**Figure 12:**
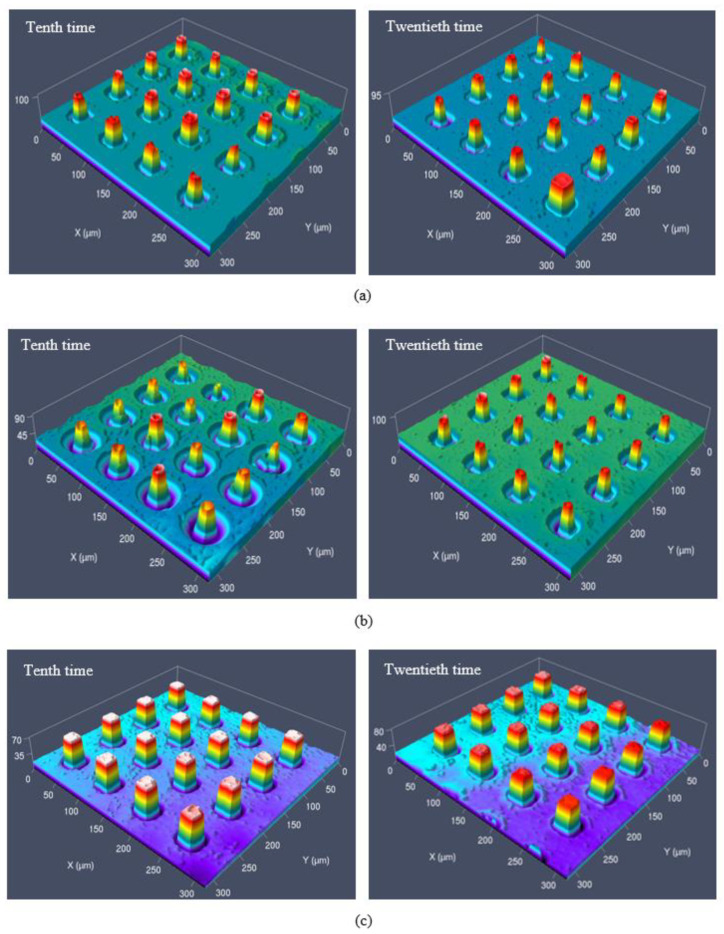
Morphology of local microstructure with demolding cycles: (**a**) DT; (**b**) DDT; (**c**) PFDT.

It could be found that when the SAMs were not deposited on the surface of the Ni mold insert, the microcolumn was relatively small, and most of the microcolumns were not fully replicated, and when the SAMs of alkyl mercaptan were deposited on the surface of Ni mold insert, the demolded parts had better reproducibility, especially PFDT SAMs, compared with the Ni mold insert without SAMs as shown in the Figure 9 and Figure 12. When the DT and DDT SAMs were deposited on the surface of the Ni mold insert, obvious defects were observed in some columns with the increase of demolding cycles; nevertheless, most of them could replicate the original morphology of the microcavity, as shown in Figure 12a,b. 

When the PFDT SAMs were deposited on the insert surface, the microcolumn showed high replication quality in the twentieth demolding cycles, as shown in Figure 12c. There was almost no concave deformation, and the necking deformation at the root of the microcolumn was small. Overall, the microcolumn had better morphology and uniformity.

**Figure 13:**
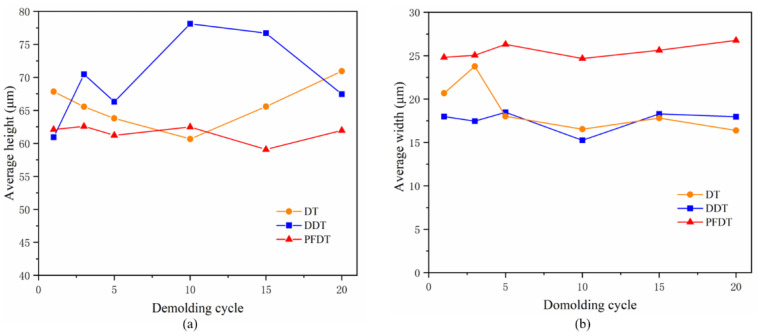
The (**a**) average height and (**b**) average width with demolding cycles.

As shown in Figure 13, when the DT and DDT SAMs were deposited on the surface of Ni mold insert, the average width was the best at the beginning. With the increase of demolding cycles, the average width gradually decreased and the microcolumn could not reproduce well. The average height defect increased gradually, which was due to the increase of adhesion between polymer and Ni mold insert because of the failure of the SAMs. When the PFDT SAMs were deposited on the surface of Ni mold insert, the average width and height of the SAMs remained good with the increase of demolding cycles.

**Figure 14:**
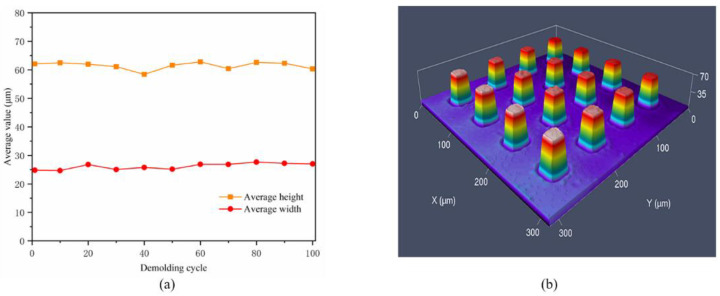
The 100th demolding cycle with the PFDT SAMs on the surface of Ni mold insert: (**a**) average height and width; (**b**) morphology of local microstructure.

It is seen that PFDT SAMs have the best wear resistance and could effectively reduce the friction and adhesion between PP and Ni mold insert, which was consistent with the friction coefficient measured by friction test. As shown in Figure 14a, even at the 100th demolding cycle, the average height and width of the microcolumn remained to maintain its uniformity. Moreover, the shape of the micron column was still fully molded and showed a high degree of replicability, as shown in Figure 14b.

## 4. Conclusions

In this work, the SAMs of alkyl mercaptan were successfully prepared on the surface of Ni insert. The results show that alkyl mercaptan could greatly reduce the surface energy of Ni insert and therefore reduce the adhesion during the demolding. Without the SAMs, the microcolumns had defects in elongation in height, depression at the top and neck shrinkage. The widths and heights of the microcolumn were obviously smaller than these of the microcavity. When the SAMs of alkyl mercaptan were deposited on the surface of Ni insert, the defects of the injection-molded columns were greatly improved. Each microcolumn had high homogeneity in three-dimensional morphology, which could reproduce the morphology of the microcavity. However, the wear resistance of the DT and DDT SAMs was relatively poor, and its effective life in microinjection molding was less than five cycles. On the other hand, the self-assembled perfluorocarbon coating had an excellent wear resistance. The microcolumn could still show high replication quality after twenty demolding cycles. When there were no SAMs on the insert surface, the width deviation of the microcolumn profile was negative, and the microstructure had a shrinkage deformation. When the alkyl mercaptan SAMs were deposited on the insert surface, the width deviation of the microcolumn was positive, and the microstructure expanded.

## Figures and Tables

**Table 1:** The basic attribute parameters of three kinds of reagents.

Name	Molecular Formula	Molecular Weight	Flash Point (°C)	Purity (%)	Boiling Point (°C)
DT	C10H22S	174	98	98	114
DDT	C10H22S2	206	171	99	171
PFDT	C10H5F17S	480	66	97	82

**Table 2:** Main process parameters for the injection molding experiment.

Mold Temperature (°C)	Melt Temperature (°C)	Injection Rate (mm/s)	Packing Pressure (MPa)	Packing Time (s)	Back Pressure (Mpa)	Cooling Time (s)
100	250	18	140	10	5	60

**Table 3:** Surface roughness of SAMs.

Type	R_q_ (nm)	R_a_ (nm)	R_max_ (nm)
No coating	1	0.724	10.4
DT	1.89	1.35	22.3
DDT	3.48	2.78	25.5
PFDT	2.14	1.69	16.7
